# Effect of collaborative depression treatment on risk for diabetes: A 9-year follow-up of the IMPACT randomized controlled trial

**DOI:** 10.1371/journal.pone.0200248

**Published:** 2018-08-23

**Authors:** Tasneem Khambaty, Christopher M. Callahan, Jesse C. Stewart

**Affiliations:** 1 Department of Psychology, University of Maryland, Baltimore County, Baltimore, Maryland, United States of America; 2 Indiana University Center for Aging Research and Department of Medicine, Indiana University School of Medicine, Indianapolis, Indiana, United States of America; 3 Regenstrief Institute, Inc., Indianapolis, Indiana, United States of America; 4 Department of Psychology, Indiana University-Purdue University Indianapolis, Indianapolis, Indiana, United States of America; Universidade Federal do Rio de Janeiro, BRAZIL

## Abstract

Considerable epidemiologic evidence and plausible biobehavioral mechanisms suggest that depression is an independent risk factor for diabetes. Moreover, reducing the elevated diabetes risk of depressed individuals is imperative given that both conditions are leading causes of death and disability. However, because no prior study has examined clinical diabetes outcomes among depressed patients at risk for diabetes, the question of whether depression treatment prevents or delays diabetes onset remains unanswered. Accordingly, we examined the effect of a 12-month collaborative care program for late-life depression on 9-year diabetes incidence among depressed, older adults initially free of diabetes. Participants were 119 primary care patients [M (SD) age: 67.2 (6.9) years, 41% African American] with a depressive disorder but without diabetes enrolled at the Indiana sites of the Improving Mood-Promoting Access to Collaborative Treatment (IMPACT) trial. Incident diabetes cases were defined as diabetes diagnoses, positive laboratory values, or diabetes medication prescription, and were identified using electronic medical record and Medicare/Medicaid data. Surprisingly, the rate of incident diabetes in the collaborative care group was 37% (22/59) versus 28% (17/60) in the usual care group. Even though the collaborative care group exhibited greater reductions in depressive symptom severity (*p* = .024), unadjusted (*HR* = 1.29, 95% *CI*: 0.69–2.43, *p* = .428) and adjusted (*HR* = 1.18, 95% *CI*: 0.61–2.29, *p* = .616) Cox proportional hazards models indicated that the risk of incident diabetes did not differ between the treatment groups. Our novel preliminary findings raise the possibility that depression treatment alone may be insufficient to reduce the excess diabetes risk of depressed, older adults.

## Introduction

Type 2 diabetes is a serious metabolic condition that is highly prevalent worldwide (9%) and has substantial consequences for individuals and for society [1]. Depression, the leading cause of disability worldwide, affects 350 million people and therefore, is also highly prevalent [2]. Findings from prospective cohort studies indicate that depression is an independent risk factor for diabetes, with risk ratios similar to well-established diabetes risk factors, including obesity, smoking, and physical inactivity [3]. Additionally, plausible biological (e.g., hypothalamic-pituitary-adrenal (HPA) axis hyperactivation, systemic inflammation) and behavioral (e.g., poor diet, physical inactivity) mechanisms underlying the depression-to-diabetes relationship have been identified [3]. A recent meta-analysis confirmed the elevated diabetes risk associated with depression, concluding that depressed adults have a 60% greater risk of developing type 2 diabetes than their nondepressed counterparts [3].

Findings from a few small intervention studies targeting depressed individuals without diabetes at baseline have shown that depression treatment improves a marker of prediabetes known as insulin sensitivity, particularly among patients whose achieved depression remission [4–6]. Although these findings are promising, only intervention studies examining incident clinical diabetes as an outcome can answer the key question of whether depression treatment prevents or delays the onset of type 2 diabetes. If such a study yields positive results, depression treatment could be pursued as a promising new approach for the primary prevention of diabetes. However, even negative results would be quite informative, as they would imply an alternate approach to reduce the risk for this highly prevalent chronic disease, for instance, comprehensive depression care in conjunction with a lifestyle intervention to address well-established diabetes risk factors (e.g., obesity). Despite the urgent need for addressing this key question, no prior study has examined incident clinical diabetes outcomes among depressed patients at risk for diabetes. Accordingly, we conducted a preliminary study examining the effects of a 12-month collaborative care program for late-life depression on the 9-year incidence of diabetes among depressed, older adults initially free of diabetes.

## Materials and methods

### Participants

We conducted a 9-year follow-up study of the Improving Mood-Promoting Access to Collaborative Treatment (IMPACT) trial, a multisite, randomized controlled trial that examined the effectiveness of collaborative care for late-life depression among depressed, older primary care patients (See [7] for detailed trial methods; ClinicalTrials.gov Identifier: NCT01561105; http://clinicaltrials.gov/ct2/show/NCT01561105). To ascertain eligibility for the trial, patients underwent a depression screen [8] and an eligibility interview [9]. Inclusion criteria for the IMPACT trial were age ≥60 years and a current major depressive disorder or dysthymia diagnosis, while exclusion criteria were: a drinking problem [10], bipolar disorder/psychosis, currently in psychiatric treatment, severe cognitive impairment [11], or at acute risk of suicide. Our follow-up study was approved by the IUPUI Institutional Review Board and the Centers for Medicare and Medicaid Services Privacy Board. Participants provided written informed consent to the IMPACT procedures, and a waiver of consent was obtained to link electronic medical record and Medicare/Medicaid data.

This study utilized data from the 235 participants enrolled at the Indiana sites of the IMPACT trial, the only cohort for whom a unique set of resources–i.e., local electronic medical record data (including death certificate data) linked with Medicare and Medicaid claims–were available. Notably, this study utilized a local electronic medical record, the Regenstrief Medical Record System (RMRS) [12], which is one of the largest and longest operating electronic medical records (earliest data from 1978). Using RMRS, we excluded 116 participants with prevalent diabetes at baseline given that the focus of this study was new-onset diabetes. Prevalent diabetes was defined as the presence of any of the following before the participant’s IMPACT enrollment date: (a) a diabetes diagnosis (ICD-9 code of 250); (b) a fasting glucose value ≥ 126 mg/dL; (c) an HbA_1c_ value ≥ 8.0%; or (d) a prescription for insulin or oral hypoglycemic medication. We used a cut point of ≥ 8.0% for HbA_1c_ rather than the American Diabetes Association’s cut point of ≥ 6.5% [13] because more recently published guidelines [14] recommend the use of a higher cut point (between 8–9%) for diagnosis among older adults who have comorbid medical conditions. We chose the more conservative cut point in this range. The final sample consisted of 119 participants.

### Treatment groups

In the IMPACT trial, participants were randomized to 12 months of the IMPACT collaborative care program or usual primary care for depression. The IMPACT intervention has been described in detail elsewhere [7, 15]. Participants in the IMPACT group worked with depression clinical specialists (DCSs) and their primary care providers to receive evidence-based depression treatment according to a stepped care algorithm that varied intervention type and intensity according to clinical needs and patient preference. Step 1 of the algorithm recommended that patients begin antidepressant medication (usually a selective serotonin reuptake inhibitor [SSRI]) or a course of Problem Solving Treatment in Primary Care (PST-PC) [16], a brief cognitive-behavioral therapy. Patients who achieved remission followed a relapse prevention plan developed by the DCS. Patients who did not respond in 8–12 weeks proceeded to Step 2 of the algorithm, which consisted of augmenting Step 1 treatment with a second antidepressant or psychotherapy or switching to another antidepressant or psychotherapy. A psychiatric consultation was initiated for patients with persistent depression. Patients who had not achieved remission after 6–10 additional weeks proceeded to Step 3, in which further medication changes, psychotherapy, hospitalization, or other mental health services were considered. Patients in the usual care group were informed of their diagnosis, were encouraged to follow-up with their primary care provider, and were followed for 12 months while they received services that were part of usual care. Providers received a letter indicating that their patient has a depressive disorder and was randomized to usual care.

### Outcome measures

Depression symptom severity was assessed at baseline and 12 months using the 20 depression items of the Symptom Checklist-90 (SCL-20) [15, 17]. The SCL-20 is a widely used outcome measure in primary care trials [18–21]. The measure has demonstrated good internal consistency in previous studies (Cronbach’s *α* = 0.84–0.86) [22, 23], as well as in the IMPACT sample recruited from the Indiana sites (Cronbach’s *α* = 0.81 at baseline and 0.91 at 12 months). In terms of validity, the SCL-20 and PHQ-9, which is an established depression measure, have been found to be moderately correlated with one another (*r* = 0.54). In addition, a 50% reduction in SCL-20 score has been shown to accurately identify 79% of patients who no longer met criteria for MDD after 12 weeks of collaborative care, suggesting that this cut point is a good indicator of change in depression status [24]. At 12 months, participants were also asked about psychotherapy and antidepressants received during the trial. Incident diabetes cases were identified using data from the RMRS, merged with claims data from Medicare and Medicaid. Incident diabetes was defined as the first occurrence of any of the following between the participant’s IMPACT enrollment date (1999–2001) and December 31, 2009: (a) diabetes diagnosis (ICD-9 code of 250); (b) a fasting glucose value ≥ 126 mg/dL; (c) an HbA_1c_ value ≥ 8.0% or (d) a prescription for insulin or oral hypoglycemic medication.

### Other variables

During the IMPACT baseline interview, patients were asked by trained lay interviewers about demographic information (age, sex, race/ethnicity) and if they had been diagnosed or treated for any of 10 common chronic medical problems in the preceding 3 years, including diabetes and hypertension [15]. Data regarding baseline smoking status and BMI were obtained through RMRS. Several indicators of smoking status were obtained, including any smoking diagnoses, yes/no markers for current smoking status, and packs-per-day information. If any of these indicators was positive, the participant received a code of ‘1’ (yes) on smoking status; otherwise, the participant received a code of ‘0’ (no). Height and weight information was also obtained from RMRS. BMI was calculated as weight in kilograms divided by the square of height in meters (kg/m^2^). Participants who endorsed use of antidepressants in the 3 months preceding the baseline interview received a code of ‘1’ (yes) on this variable; otherwise, participants received a code of ‘0’ (no).

#### Data analyses

Prior to conducting any hypothesis-testing analyses, chi-square tests (for categorical variables) and independent samples *t* tests (for continuous variables) were conducted to compare baseline characteristics between patients in the IMPACT and usual care groups. Additionally, a Cohen’s *d* effect size was calculated to quantify the effect of the IMPACT intervention on change in SCL-20 score. To test our primary hypothesis, Cox proportional hazard regression models were constructed [25]. Cox models yield hazard ratios (*HR*) as the primary statistic. For this study, *HR*s estimated the relative likelihood of incident diabetes in the IMPACT group versus control group. Patients were censored at their date of death or at the end of the follow-up period (December 31, 2009). The primary Cox model included the randomization status variable (IMPACT vs. usual care) as the only independent variable (no covariates). To supplement these primary analysis, Kaplan-Meier survival curves were constructed to illustrate the time from enrollment to incident diabetes for each treatment group. A second Cox model was constructed to include baseline age, sex, race/ethnicity, hypertension, smoking, and BMI variables in addition to the randomization status variable (fully-adjusted analyses). Analyses were performed using SAS statistical software, version 9.3 (SAS Institute, Cary, NC).

We conducted two sets of sensitivity analyses. First, reran Cox models after modifying our incident diabetes definition. The alternative definitions were: (a) Any diagnosis: ICD-9 code of 250 (b); Any lab value: fasting glucose value ≥ 126 mg/dL OR HbA1c value ≥ 8.0%; (c) Any diabetes medication: insulin or oral hypoglycemic medication. Second, we evaluated whether the effect of collaborative depression treatment on diabetes was influenced by change in depressive symptoms during the trial. To do so, we created an SCL-20 change score (subtracting the 12-month SCL-20 score from the baseline score) and added it to the unadjusted Cox model that only included the randomization variable.

## Results

### Effect of the IMPACT intervention on depression outcomes and care

For our sample of 119 depressed, older adults who were free of diabetes at baseline (IMPACT: *n* = 59, usual care: *n* = 60), independent sample *t* tests and chi-square tests revealed no group differences in baseline age, sex, race/ethnicity, hypertension, and smoking (Table 1). However, baseline BMI of the IMPACT group was higher than that of the usual care group (31.3 vs. 27.7 kg/m^2^; *p* = .024). At post-treatment (12 months), IMPACT patients exhibited greater reductions in SCL-20 score than usual care patients (*p* = .024), with a treatment effect size (*d* = 0.43) in the medium range [26], suggesting greater improvement in depressive symptoms in the IMPACT vs. usual care group. IMPACT patients were more likely than usual care patients to have received psychotherapy (60% vs. 17%, *p* < .001) but not antidepressant medication (73% vs. 57%, *p* = .064) during the trial (Table 1).

**Table 1:** Characteristics of participants by treatment group.

Characteristic	Total Sample (*N* = 119)	IMPACT (*n* = 59)	Usual Care (*n* = 60)	*p* value
*Baseline Demographic Factors*				
Age, mean (SD)	67.2 (6.9)	66.7 (6.5)	67.7 (7.3)	.428
Male, %	23.5	20.3	26.7	.416
African-American, %	41.2	39.0	43.3	.630
Height (inches), mean (SD) ^Ϯ^	64.6 (3.5)	64.2 (2.9)	65.0 (3.9)	.221
Weight (pounds), mean (SD) ^Ϯ^	175.1 (52.3)	183.3 (57.3)	167.0 (46.0)	.089
*Baseline Diabetes Risk Factors*				
Hypertension, %	73.1	72.9	73.3	.956
Smoker, %	36.1	32.2	40.0	.376
Body-Mass Index (kg/m^2^), mean (SD)	29.5 (8.5)	31.3 (9.6)	27.7 (7.0)	.024
*Baseline Depression Variables*				
MDD Only, %	12.6	11.9	13.3	.809
Dysthymia Only, %	33.6	33.9	33.3	.948
MDD and Dysthymia, %	53.8	54.2	53.3	.921
SCL-20 Score, mean (SD) (range: 0–4)	1.4 (0.5)	1.3 (0.6)	1.5 (0.5)	.121
Antidepressant Use in Past 3 Months, %	50.4	52.5	48.3	.646
*Depression Outcomes and Care Variables*				
SCL-20 Change, mean (SD) (*N* = 111)	-0.13 (0.7)	-0.3 (0.7)	0.0 (0.7)	.024
Antidepressants during the trial, %	64.7	72.9	56.7	.064
Psychotherapy during the trial, %	37.8	59.3	16.7	< .001

*Note*. *N* = 119 except where indicated. Independent samples *t* tests were used to compare groups on age, body mass index, baseline SCL-20 score, and SCL-20 change. All other group comparisons were made using chi-square tests. IMPACT = Improving Mood-Promoting Access to Collaborative Treatment. MDD = major depressive disorder. SCL-20 = Symptom Checklist-20.

^Ϯ^SI conversions: To convert inches to centimeters, multiply by 2.54; To convert pounds to kilograms, multiply by 0.45

### Effect of the IMPACT intervention on incident diabetes

**Fig 1:**
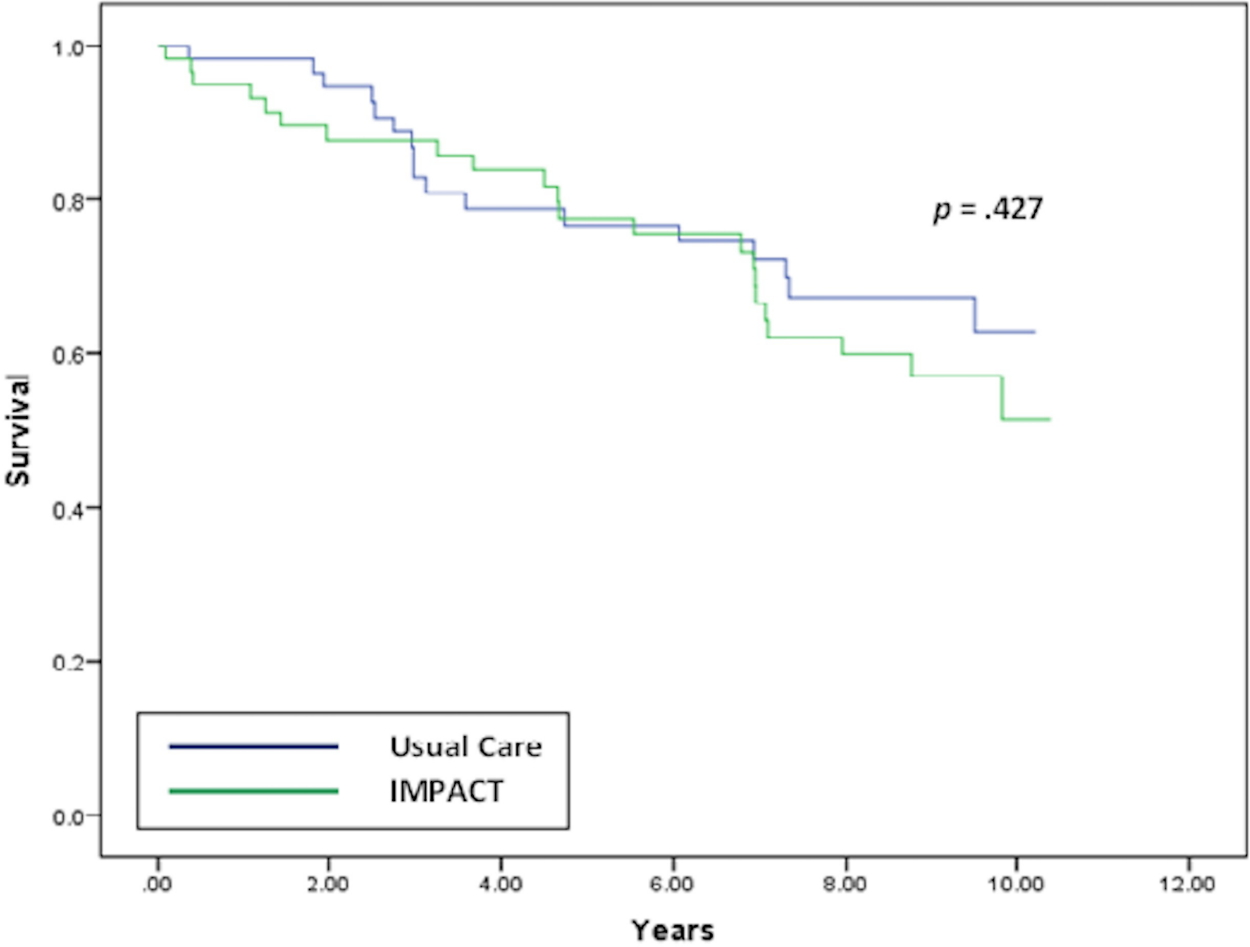
Time to incident diabetes for IMPACT participants. Kaplan-Meier survival curves depicting time to incident diabetes (diabetes diagnosis, positive laboratory value, or diabetes medication prescription) among depressed, older adults initially free of diabetes randomized to a 12-month collaborative care program for depression (*n* = 59) or usual care (*n* = 60). IMPACT = Improving Mood-Promoting Access to Collaborative Treatment.

Thirty-nine incident clinical diabetes cases (33%) were identified during the 9-year follow-up period. The rate of incident diabetes in the IMPACT group was 37% (22/59) versus 28% in the usual care group (17/60). Fig 1 displays the Kaplan-Meier survival curves illustrating the time to incident diabetes for both treatment groups. A log-rank test indicated that there was no group difference in incident diabetes (*χ*^*2*^ = .63, *p* = .427). Cox proportional hazards models confirmed this finding. The unadjusted Cox model indicated that the risk of incident diabetes did not differ between the collaborative care and usual care groups (*HR* = 1.29, 95% *CI*: 0.69–2.43, *p* = .428), as did a Cox model adjusting for baseline age, sex, race/ethnicity, hypertension, smoking, and BMI (*HR* = 1.18, 95% *CI*: 0.61–2.29, *p* = .616). In the adjusted model, only baseline BMI predicted incident diabetes (*HR* = 1.05, 95% *CI*: 1.00–1.10, *p* = .027).

### Sensitivity analyses

When we considered alternative definitions of incident diabetes, we found that there was some variability in the rate of incident diabetes across these definitions, ranging from 11 events (IMPACT = 7; usual care = 4) for the any medication outcome to 33 events (IMPACT = 19; usual care = 14) for the any lab value outcome (see Table 2). There was also variability in the treatment group differences in incident diabetes rates, with the lowest and highest treatment group differences observed for the any medication outcome (IMPACT = 11.9%; usual care = 6.7%) and the any diagnosis outcome (IMPACT = 22.0%; Usual Care = 11.7%), respectively. Overall, collaborative care patients continued to have a similar risk of incident clinical diabetes over nine years as usual care patients in unadjusted and fully-adjusted analyses (see Table 2). Across all analyses, low event rates and large confidence intervals render interpretation difficult but suggest that the relationship between depression treatment and incident diabetes events does not vary by the type of incident diabetes definition used.

**Table 2:** Results of Cox proportional hazard regression models examining treatment group as a predictor of incident diabetes–alternative definitions of incident diabetes.

Diabetes Outcome	Total Sample (N = 119)	IMPACT (*n* = 59)	Usual Care (*n* = 60)	Treatment Group (IMPACT vs. Usual Care)		
	Events (%)			*HR*	95% *CI*	*p* value
Any Diagnosis §	20 (16.8%)	13 (22.0%)	7 (11.7%)	1.75	0.69–4.46	.237
Fully-Adjusted ‡				1.66	0.61–4.51	.319
Any Lab Value *	33 (27.7%)	19 (32.2%)	14 (23.3%)	1.31	0.66–2.61	.446
Fully-Adjusted ‡				1.11	0.54–2.28	.779
Any Medication Ϯ	11 (9.2%)	7 (11.9%)	4 (6.7%)	1.76	0.52–6.02	.366
Fully-Adjusted ‡				1.82	0.38–8.67	.451

*Note*. *N* = 119. *HR* = hazard ratio. *CI* = confidence interval. ICD-9 = International Classification of Diseases-9^th^ Revision. IMPACT = Improving Mood-Promoting Access to Collaborative Treatment. HbA_1c_ = Hemoglobin A_1c_

§ Defined as an ICD-9 code for diabetes.

‡ Adjusted for age, sex, race/ethnicity, hypertension, smoking status, and body mass index.

*Defined as a fasting glucose value ≥ 126mg/dL OR an HbA_1c_ value ≥ 8.0%.

Ϯ Defined as a prescription for diabetes medication (insulin or oral hypoglycemic medication).

Finally, when we adjusted our primary analyses for change in depressive symptoms over the 1-year intervention period, the hazard ratio for the treatment effect on incident diabetes was not meaningfully altered (HR = 1.13, 95% CI: 0.59–2.18, p = .71), and change in depressive symptoms did not predict incident diabetes (HR = 0.81, 95% CI: 0.49–1.34, p = .41).

## Discussion

Consistent with results of the parent IMPACT trial [7] and other depression trials involving older adults [27], we found that depressed, older primary care patients randomized to collaborative depression care exhibited significantly greater reductions in depressive symptoms than those randomized to usual care. Despite these improvements, collaborative care patients had a similar risk of incident clinical diabetes over nine years as usual care patients, even when alternative definitions of incident diabetes were considered.

Our preliminary findings do not align with results of prior intervention studies with non-diabetic samples, assessing insulin sensitivity outcomes. In two previous studies [4, 6], depressed, non-diabetic patients who were given either tricyclic or SSRI antidepressants and who achieved depression remission showed improved insulin sensitivity over a 5- to 8-week period. Of note, in the larger of these two studies, depression remission was positively associated with only one of three diabetes outcomes examined (insulin level after a glucose challenge, but not fasting insulin or glucose levels). In another study [5], Okamura et al. reported that depressed, non-diabetic patients who received either tricyclic or tetracyclic antidepressants showed improvement in insulin sensitivity from pre- to post-treatment, as assessed by oral glucose tolerance tests. However, because all three of these studies did not have a control group, it is possible that factors other than the depression interventions were responsible for the observed improvements in insulin sensitivity. In contrast, our results are consistent with those of Kauffman and colleagues [28], who showed that 8 weeks of SSRI treatment did not produce improvement in insulin sensitivity, as measured by oral glucose tolerance tests. Yet, even these results cannot easily be compared to our results because of their dissimilar sample of 32 depressed and nondepressed, euglycemic women of reproductive age. As is evident by these studies, there is a dearth of rigorous research literature in which the effect of depression treatment on diabetes-related outcomes is examined in samples of depressed patients initially free of diabetes.

There are at least two possible explanations for nonsignificantly elevated diabetes risk in the IMPACT versus the usual care group. One possibility is that the higher baseline BMI of the IMPACT group contributed to the elevated rate of incident diabetes. Specifically, this may have led to an increase in the degree of insulin resistance and the prevalence of pre-diabetes in the IMPACT group. Consequently, a higher percentage of patients in this arm would transition to diagnosed, clinical diabetes during the follow-up. The BMI-adjusted analyses provide partial support for this notion, given that BMI was a significant predictor of incident diabetes, and adjustment for BMI attenuated the hazard ratio. A second possibility is that IMPACT patients had greater contact with the healthcare system due to the intervention and therefore, were more likely to be referred for other health services during the 9-year follow-up period. As a result, this group may have been followed more closely and had more opportunities for detection of new-onset diabetes than the usual care group.

There are also three leading explanations for the null effect of collaborative depression care on diabetes risk. The first is the older age of the sample. Because insulin resistance increases with age [29], its severity in the IMPACT sample, despite the absence of diagnosed diabetes, was likely high. In addition, there was a high prevalence of diabetes risk factors (e.g., BMI) in the sample at baseline (see Table 1). Together, the older age and, therefore, severity of insulin resistance of this cohort, in conjunction with its high baseline diabetes risk factors status, may have overridden any effect of depression treatment on diabetes incidence. Nevertheless, whether or not depression treatment alone lowers diabetes risk in middle-aged and younger adults is an open question. Given the older age of our sample, many diabetes cases, perhaps especially those that were depression related, likely developed prior to enrollment in the IMPACT Trial and these patients are not included in our sample. The second is potential depression treatment improvement in the usual care group. As Table 1 demonstrates, 57% and 17% of individuals in the usual care arm received antidepressant medication and psychotherapy, respectively. It is possible that without this relatively high level of depression treatment, depressive symptom severity in this group may have worsened over time. Thus, due to a greater number of usual care patients being treated for depression, group differences in depressive symptom improvement, and subsequently, incident diabetes, were likely reduced. The third is the high rate of antidepressant exposure in both groups and the potential diabetogenic effect of these medications. As noted in Table 1, 73% and 57% of the IMPACT and usual care groups, respectively, received antidepressant medication during the intervention. Studies demonstrate that some antidepressant medications are associated with and increased risk of type 2 diabetes through various mechanisms, including appetite promotion and weight gain [30–32]. Taken together, increased antidepressant medication use in both groups may have resulted in the lack of treatment group differences in incident diabetes we observed. However, it should be noted that because other studies demonstrate no effects or weight loss as a result of antidepressant use [33, 34], the current literature linking antidepressant medication and diabetes appears to be inconclusive.

While our study is unique and begins to address an important public health topic, its findings are preliminary and should be interpreted in light of the following limitations. First, our analyses were observational in nature because the IMPACT trial was not designed to examine our specific question. For example, randomization was not stratified by baseline diabetes, and incident diabetes was not a pre-specified outcome. While patients without diabetes were equally distributed across the treatment groups, and a strong theoretical rationale was present, only a prospective randomized controlled trial specifically designed to test the study hypotheses would allow for definitive conclusions to be drawn. Second, while we had a strong rationale for using a higher cut point for HbA_1c_ (8.0%) given the demographic and clinical characteristics of our sample [14], an HbA_1c_ cut point of > 6.5% is a more commonly accepted criterion for diabetes diagnosis [13]. Thus, future studies may consider comparing our findings with those using this and other criteria for HbA_1c_. Finally, our analyses were underpowered to fully test the study question. Consequently, we consider our results to be preliminary and in need of replication in future intervention studies.

This study begins to address a key clinical issue, as reducing the elevated diabetes risk of depressed individuals is imperative, and begins to fill an important knowledge gap, as no study has examined the effect of depression treatment on incident clinical diabetes. Our findings raise the possibility that depression treatment alone may be insufficient to reduce the excess diabetes risk of depressed adults. Although our findings are preliminary, they remain valuable for hypothesis generation and for informing the design of future intervention studies. The main objective of these future trials will be to determine whether depression treatment lowers diabetes risk and whether any treatment effects on diabetes outcomes are mediated by depression outcome or care variables. If on the other hand, these adequately-powered, well-designed and executed studies determine that depression treatment does not lower diabetes risk, then other approaches for reducing the elevated diabetes risk of depressed patients need to be identified. Specifically, if our results are replicated, it would suggest the need for an integrated biopsychosocial treatment program that simultaneously intervenes on depression and the putative mechanisms underlying the depression-diabetes relationship in order to produce clinically meaningful reductions in incident diabetes in this population.
